# Development of a simple and quick immunochromatography method for detection of anti-HPV-16/-18 antibodies

**DOI:** 10.1371/journal.pone.0171314

**Published:** 2017-02-03

**Authors:** Fumiko Endo, Tsutomu Tabata, Daichi Sadato, Machiko Kawamura, Noriyuki Ando, Keisuke Oboki, Masako Ukaji, Kaoru Kobayashi, Yukuharu Kobayashi, Tomoaki Ikeda, Futoshi Shibasaki

**Affiliations:** 1 Department of Molecular Medical Research, Tokyo Metropolitan Institute of Medical Science, 2-1-6 Kami-Kitazawa, Setagaya-ku, Tokyo, Japan; 2 Department of Obstetrics and Gynecology, Faculty of Medicine, Mie University, 2–174 Edobashi, Tsu-city, Mie, Japan; 3 Department of Applied Biological Science, Faculty of Science and Technology, Tokyo University of Science, 2641 Yamazaki, Noda, Chiba, Japan; 4 Department of Pediatrics, Tokyo Metropolitan Cancer and Infectious Diseases Center Komagome Hospital, 3-18-22 Honkomagome, Bunkyo-ku, Tokyo, Japan; 5 Josei-Kokoro-Clinic, 1-1-9 Machiya, Arakawa-ku, Tokyo, Japan; 6 ADTEC Co., LTD. 1693–6 Yokkaichi, Usa-City, Oita, Japan; Fondazione IRCCS Istituto Nazionale dei Tumori, ITALY

## Abstract

Immunochromatography (IC) is widely used to detect target molecules in biological fluids. Since this method can be performed without a special technique or device, IC is a convenient way to assess the existence of antibodies or pathogens such as viruses and bacteria, simply and quickly. In this study, we established an IC method to detect serum antibodies against oncogenic human papillomavirus (HPV)-16 and HPV-18 L1 proteins using recombinant L1 proteins produced by silkworms as antigens. Infection of oncogenic HPVs is a major risk factor of cervical cancer, which is one of the most common cancers in women worldwide. We first measured blood sera of two groups by magnetic beads enzyme-linked immunosorbent assay (MB-ELISA). For the first group, sera were collected prospectively from young women who planned to receive HPV vaccination. The second group consisted of children under 20 years of age, non-vaccinated healthy women, vaccinated healthy women, dysplasia, cervical intraepithelial neoplasia III, and cervical cancer patients. We confirmed that standard vaccination doses significantly increased serum HPV antibody concentrations, and the level was sustained at least more than 30 months after vaccination. In contrast, an increase in antibody concentration was not observed in patients with precancerous cervical changes and cervical cancer. We next measured the samples in both groups using the IC method we originally developed, and found that the measurement values of IC highly correlated with those of MB-ELISA. The simple and quick IC method would be a useful tool for rapid monitoring of L1 specific antibody levels in a non-laboratory environment. With less than one drop of serum, our IC can easily detect serum HPV-16/-18 antibodies within 15 minutes, without the need for electronic devices or techniques.

## Introduction

Human papillomavirus (HPV) has a non-enveloped capsid and a circular double stranded DNA genome. The global prevalence of HPV infection is estimated at around 11–12% [1,2]. To date, genomic sequencing analysis for HPV can distinguish over 100 types. Among them, at least 15 are oncogenic such as HPV-16 and HPV-18 [3]. Cervical cancer, which is one of the most common cancers in women worldwide, is the most important HPV-associated cancer [4]. Persistent infection with HPV in the genital tract ultimately leads to a high incidence of cervical cancer [5,6].

The first two HPV vaccines on the market, the bivalent vaccine by GlaxoSmithKline (Cervarix^®^) containing HPV-16 and -18 antigens, and the quadrivalent vaccine by Merck (Gardasil^®^) containing HPV-6, -11, -16, and -18 antigens, have been well studied for almost a decade [7,8]. HPV-16 and HPV-18 are highly oncogenic and expressed in 70% of invasive cervical cancer [9]. Both vaccines induce positive seroconversion for serum anti-HPV antibodies (Abs), and have long term efficacy to prevent HPV infection and changes in the precancerous cervical epithelium antecedent to the development of cancer [7,8,10–14].

The levels of serum anti-HPV Abs is one of the significant indicators to estimate the efficacy of HPV vaccination. In the case of natural infection of HPVs, specific Ab responses are induced against the HPV antigens, and these Abs are able to protect partially but not completely against subsequent infection [15]. In vaccinated individuals, it is important to investigate the immunogenicity of vaccines based on specific Ab responses. This is also necessary to determine the appropriate dose and protocol of vaccination[16], and the effective threshold of serum Ab concentration to protect against HPV infection [17,18]. Vaccination by Cervarix^®^, a bivalent vaccine, was reported to sustain the high levels of serum anti-HPV Abs at 113 months post vaccination [11]. With Gardasil, a quadrivalent vaccine, the level of anti-HPV Ab was also reported to be maintained at high levels 108 months post vaccination [19]. This clearly suggests that specific Abs against both vaccines are likely to last for long periods of time. Correspondingly, the prospective large scale cohort studies show a long term efficacy with protection from precancerous change in the genital organs, including the uterine cervix [20–25].

Available prophylactic HPV vaccines utilize the recombinant HPV major capsid L1 protein as an antigen that forms capsid-like multimers by intrinsic activity, which is referred to as a virus-like particle (VLP) [26,27]. VLPs have also been used for the detection of HPV L1-specific Abs. Anti-HPV L1 Ab levels in serum have been measured by enzyme-linked immunosorbent assay (ELISA), competitive Luminex immunoassay (cLIA) [28], and an *in vitro* neutralization assay called pseudovirion-based neutralization assay (PBNA) [17,29,30]. The cLIA and PBNA measure very restricted anti-VLP Abs (i.e. HPV type-specific, neutralizing), which are a subset of the total immune response to multivalent HPV VLP vaccination. As a result, cLIA and PBNA might underrepresent the VLP-induced protective whole Ab clones elicited by vaccination with L1 VLPs [31]. The titer of total anti-L1 Ab measured by ELISA highly correlates with that of HPV type-specific Abs by cLIA and that of neutralizing Ab by PBNA [31–33]. These suggest that the level of total anti-L1 Ab is a reliable surrogate marker for monitoring the efficacy of HPV vaccination.

In this study, we developed an immunochromatography (IC) method to measure serum anti-HPV-16/-18 Ab concentrations, in addition to our originally developed magnetic beads ELISA (MB- ELISA). Our IC method successfully measured Abs in the serum of vaccinated individuals and a cohort including cervical cancer-related entities, with correlated results measured by MB-ELISA.

## Materials and methods

### Ethics statement

This study was approved by the Ethics Review Board of Tokyo Metropolitan Institute of Medical Science (IRB#17–13), Mie University (IRB#2388), and Tokyo Metropolitan Cancer and Infectious Diseases Center Komagome Hospital (IRB#1061). All serum samples were obtained from women who provided signed informed consent. In the case of children, we used the surplus specimen, which was collected for their treatment with signed informed consent by their parents.

### Reagents

TMB peroxidase substrate and stop solution were purchased from Kirkegaard & Perry Laboratories, Inc. (Gaithersburg, MD, USA). One-Step NBT/BCIP (nitro-blue tetrazolium and 5-bromo-4-chloro-3'-indolyphosphate) was purchased from Thermo Fisher Scientific Inc. (Rockford, IL, USA). ZyMAX™ horseradish peroxide (HRP)-labeled goat polyclonal anti-human IgG (H + L) was purchased from Life technologies (Camarillo, CA, USA). Human papillomavirus vaccines, Cervarix^®^ (including recombinant human HPV-16 and -18 L1 proteins; GlaxoSmithKline Biologicals, Rixensart, Belgium) and Gardasil^®^ (including recombinant HPV- 6, -11, -16, and -18 L1 proteins; Merck, White Station, NJ, USA), were purchased from the manufacturers. World Health Organization (WHO) international standard HPV-16 (NIBSC code: 05/134, 5 units per ampoule) and HPV-18 antibodies (NIBSC code: 10/134, 8 units per ampoule) were purchased from NIBSC (London, UK). Normal anonymized human female sera, which were collected under an IRB approved protocol for de-identified, were purchased from Complex Antibodies, Inc. (Margate, FL, USA) and were used as a control serum which showed no immunoreactivity against the Cervarix^®^ antigen based on our prior ELISA.

### HPV-16 and HPV-18 L1 production and purification

HPV-16 and HPV-18 L1 proteins were expressed in the silkworm-Baculovirus system produced by ProCube^®^ (Sysmex Corporation, Kobe Japan). HPV-16 L1 (GenBank Accession No. GQ423063) and HPV-18 L1 (GenBank Accession No. AY383628) genes were synthesized by Integrated DNA Technologies, Inc (Coralville, IA, USA). These genes were then ligated into pDock-His_pM01 vectors to obtain recombinant transfer vectors. This transfer vector was co-transfected with *Bombyx mori* nucleopolyhedrovirus (BmNPV) DNA [34] into a *B*. *mori*-derived cell line (BmN) [35]. After 7 days of incubation, the recombinant baculovirus was injected into the silkworm pupae. Six days after infection, the pupae were frozen and homogenized with buffer A (20 mM Tris-HCl, 150 mM NaCl, 1 mM EDTA, 1 mM EGTA, 10% Glycerol, 10 mM Benzamidine, 1 mM PMSF, 1 mM DTT, phenylthiourea, pH 8.0). The homogenized solution was centrifuged to separate the supernatant and precipitate using an ultracentrifuge (100,000 × *g*) for 1 h at 4°C. The supernatant was applied to Dock Catch Resin (Sysmex Corporation), and both proteins were eluted with buffer B (25 mM Tris-HCl, 250 mM NaCl, 10% Glycerol, 5 mM EGTA, pH 8.0). The purified proteins were concentrated with Amicon Ultra-4 30 k units (Merck KGaA, Darmstadt, Germany Millipore, Co Ltd. USA).

### ELISA

F96 Maxisorp Nunc-Immuno plates (Themo Fisher Scientific, MA, USA) were coated with purified HPV-L1 recombinant proteins or Cervarix^®^ in 50 mM carbonate buffer (pH 9.6) at 4°C overnight. After blocking with PBS containing 1% BSA (Sigma-Aldrich, MO, USA), 1,000-fold diluted two rabbit polyclonal antisera to Cervarix^®^, was added and incubated at room temperature (r.t.) for 1 h. After washing the wells 3 times with PBS containing 0.1% Tween 20 (Sigma-Aldrich), HRP-conjugated anti-rabbit IgG (Southern Biotech, AL, USA) was added and incubated at room temperature (r.t.) for 1 h. After washing the wells 3 times, o-phenylenediamine dihydrochloride (Sigma-Aldrich) was added to the wells and incubated for 5 min at r.t., followed by the addition of 1 M H_2_SO_4_. The absorbance was measured at 490 nm using the ARVO X5 (Perkin Elmer, MA, USA).

### Western blotting

Proteins obtained from pupae infected with recombinant baculovirus containing HPV-16 L1 or HPV-18 L1 were analyzed by western blotting according to the method of Towbin *et al*. [36]. The proteins were separated by SDS-PAGE using a 7.5% acrylamide gel, and transferred to a nitrocellulose membrane at 80 V for 1 h. The membrane was incubated with anti-dock-tag mouse monoclonal Ab (#KAD006, Sysmex) diluted 1:1,000, anti-His-tag mouse monoclonal Ab (D291-3, MBL) diluted 1:5,000, or rabbit antiserum diluted 1:1,000 in phosphate-buffered saline containing 0.05% Tween 20 (PBST) and 2% skim milk. The membrane was then incubated for 30 min with anti-mouse IgG alkaline phosphate-conjugated Ab (Promega, Madison, WI, USA) diluted 1:3,000 or anti-rabbit IgG alkaline phosphate-conjugated Ab (Promega) diluted 1:3,000. Antibody binding was visualized using the One-Step NBT/BCIP (Thermo Fisher Scientific).

### Magnetic Beads ELISA (MB-ELISA)

MB-ELISA was performed as previously described [37] with the modification of using 96-well U-bottomed microplates (Cat. No. 650001; Greiner Bio-One Co., Ltd. Tokyo, Japan). In each well, 4 pg of HPV-16 L1 or HPV-18 L1 recombinant protein conjugated to the tosylactivated Dynabeads M-280 (5% slurry) (Invitrogen Dynal AS, Oslo, Norway) were mixed in 50 μL of assay buffer (450 mM NaCl, 50 mM NaH_2_PO_4_-Na_2_HPO_4_, 0.05% Tween 20, 10% goat serum, pH 7.4). Next, 50 μL of each serum sample diluted at 1:100 in assay buffer was then added. Antigen-free wells were set as negative controls. After incubation for 2 hrs, the beads were captured on a 96-well Magnetic-Ring Stand (Cat. No. AM10050; Applied Biosystems, Foster City, CA) for 5 min, and washed 4 times with wash buffer (0.05% Tween-20, 0.5 M NaCl, and 20 mM Tris-HCl, pH 7.4). After washing, HRP conjugated-goat anti-human IgG (H + L) Ab, diluted 1: 5,000, was added and incubated for 30 min. The plate was then washed 4 times with the wash buffer. The reaction was started by adding Sure Blue TMB microwell peroxidase substrate, and the absorbance was measured at 450 nm using the microplate reader Benchmark (BIO-RAD, Hercules, CA, USA). All reactions were performed at room temperature. To correct the difference of OD value among plates, we employed an in-house standard sample for every measurement.

### Standardization of the assay serum for the MB-ELISA

To compensate for the variation in ELISA values among plates, we used one vaccinated sample that demonstrated high Ab concentrations of both HPV-16 and -18 as a reference for every assay. Moreover, to provide objectivity, WHO international standard HPV-16 antibodies (code: 05/134) and HPV-18 antibodies (code: 10/140) were used (National Institute for Biological Standards and Control, Hertfordshire, UK). These WHO standards were set as 1, 0.5, 0.25, and 0.125 U/mL and MB-ELISA was performed. The linear standard curves and the linear equations were obtained by plotting values (x-axis; WHO standard, y-axis; OD). The R^2^ value for HPV-16 was 0.994 and 0.9801 for HPV-18. Based on these equations, we calculated the international unit of our in-house reference serum: the values were HPV-16; 197 U/mL, and HPV-18; 138 U/mL. Using these values, OD values were converted to WHO international units (IU/mL) by comparing to an in-house reference serum in addition to the plate compensation.

### Immunochromatography

IC chips were prepared as previously described, with some modifications [37]. Briefly, HPV-16 or HPV-18 antigens were immobilized on the IC membrane test line (Test line: T). On the control line (Control line: C), anti-goat IgG Ab was immobilized to determine if the IC test was carried out accurately. The protocol is as follows: less than 20 μL of serum samples diluted with the dilution buffer (50 mM Tris-HCl, pH 7.2, 150 mM NaCl, 1% Triton X-100) was added to the IC chip in a dropwise manner. Alkaline phosphatase (AP) labeled anti-human IgG in the developing buffer (50 mM Tris-HCl, pH 7.2, 150 mM, NaCl, 1% Trition X-100, 1 mM MgCl_2_) was provided by breaking through the small pouch. The dry AP substrate (BCIP), fixed on the membrane, was mixed with conjugation buffer after opening the reservoir. Anti-HPV antibodies in serum samples were captured by the immobilized HPV-16 or HPV-18 antigen on the IC membrane and were detected by a color change indicating an enzymatic reaction. Scores of the color strength was leveled from 0 (no color) to 8 (maximum density) by visual inspection according to the control color sample card.

### Statistical analyses

Differences were evaluated by Kruskal-Wallis one-way ANOVA followed by Dunn’s post hoc multiple comparison in indices of clinical samples. Pearson’s correlation coefficient was used to assess correlations between HPV-16 and HPV-18 in MB-ELISA. Spearman’s correlation coefficient was used to assess correlations between IC and ELISA. Data are expressed as means ± standard deviation. Statistical analysis was performed by GraphPad Prism (GraphPad Software Inc., La Jolla, CA, USA) and SigmaPlot (Systat Software Inc., San Jose, CA, USA).

## Results

### Recombinant proteins of HPV-16 L1 and HPV-18 L1 and HPV type-specific immunoreactivities

**Fig 1:**
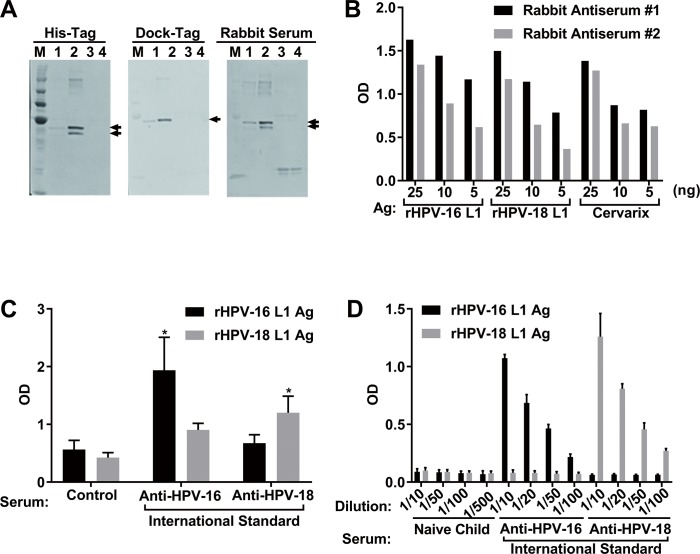
Preparing HPV-16L1/-18L1 antigens and their immunoreactivities. **A.** HPV-16 L1/HPV-18 L1 recombinant proteins obtained in this study were detected by western blot. In all membranes, line 1: Dock-Tag purified pupae infected with HPV-16 L1, line 2: Dock-Tag purified pupae infected with HPV-18 L1, line 3: non-infection pupae, and line 4: non-recombination viral infection pupae. **B.** Purified HPV-16 L1/HPV-18 L1 recombinant proteins or Cervarix^®^ were captured on plates with 25, 10, 5 ng as antigens and direct ELISA was performed. Cervarix^®^ contains HPV-16/-18 VLP and was used as a positive control. As a primary Ab, antisera of rabbit #1 (black bar) and rabbit #2 (gray bar) were used. **C.** Direct ELISA results showed HPV type-specific immunoreactivities with relatively high backgrounds by measuring with the international standard serum for HPV-16 and HPV-18. We used a control serum that was negative to HPV Ags by validation using ELISA with Cervarix^®^ Ag (**S2 Fig**). ELISA was performed twice independently in triplicate. Asterisk (*) indicates a significant difference from the control serum (*p* < 0.05). **D.** MB-ELISA showed higher type-specific immunoreactivity than the direct ELISA. Values are means of triplicate results, and error bars show standard deviations.

To detect Abs against HPV-16 L1 and HPV-18 L1 in serum from vaccinated individuals, these genes were cloned and then expressed in the silkworm-baculovirus system as described in the Materials and Methods section (**S1 Fig**). The expression of dock- and His-tagged HPV-16 L1 and HPV-18 L1 recombinant proteins was confirmed by western blotting (**Fig 1A**).

We also confirmed these antigens by ELISA using two independent polyclonal rabbit antisera immunized with Cervarix^®^. Corresponding to the quantity of the antigen, a dose-dependent absorbance change was observed (**Fig 1B**). We next checked their immunoreactivities by using the WHO HPV type-specific serums in ELISA (**Fig 1C**). Both antigens showed type-specific immunoreactivity, however, it was likely that non-negligible backgrounds were observed. To reduce the backgrounds, we employed magnetic beads method as previously described [37]. The results by MB-ELISA showed the type-specific immunoreactivities with a greater signal/background ratio than the direct ELISA (**Fig 1D**).

### Time-course analysis of anti-HPV-16 L1 and anti-HPV-18 L1 Ab in individual serum after vaccination

**Fig 2:**
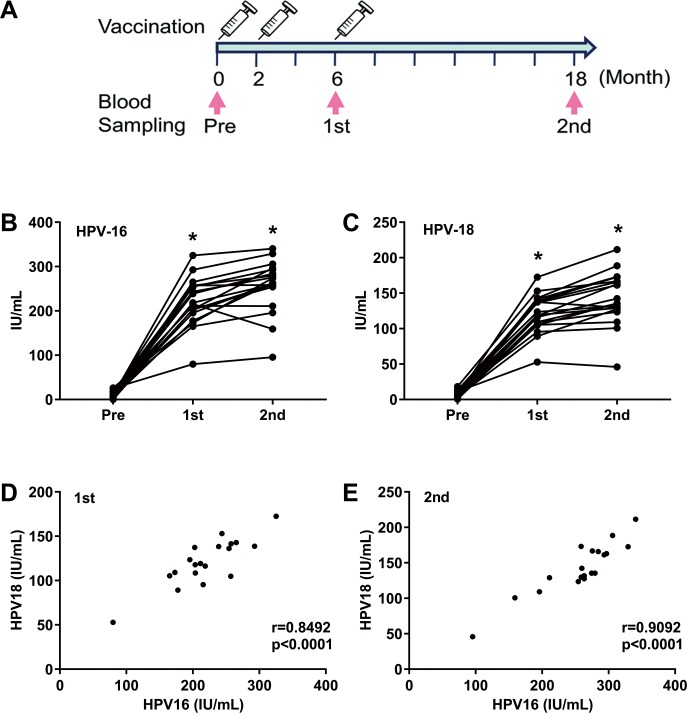
Change in HPV-16/-18 Ab levels after vaccination. **A.** A schema of the vaccination and blood sampling protocol. HPV vaccination (Gardasil^®^) was provided at 0, 2, and 6 months, and blood sampling was conducted at 0 (pre), 6 (1st), and 18 (2nd) months. **B and C.** The change in serum HPV-16 (left) and HPV-18 (right) Ab concentrations of each subject was observed at the 1st and 2nd time-point. Asterisk (*) represents p < 0.001, one-way ANOVA. **D** and **E**. Correlation between anti-HPV-16 Ab (x-axis) and anti-HPV-18 Ab (y-axis) at 1st (6 months, left) and at 2nd (18 months, right) collection time points.

We first examined whether the anti-HPV-16 L1 and anti-HPV-18 L1 Ab concentrations increased in serum after vaccination in individuals who did not receive previous HPV vaccination. Twenty healthy women were enrolled (**Table 1**) and we prospectively collected their sera from peripheral blood at three time-points including at pre-vaccination and at two time-points (6, 18 months) post vaccination (**Fig 2A**).

**Table 1:** List of vaccines and time-course sample collection.

Sample No.	Age*	Date (y.m.d)					
		Vaccination			Blood collection		
		#1	#2	#3	Pre	1st	2nd
1	12	2012.06.01	2012.08.25	2012.12.08	2012.06.01	2012.12.08	2013.12.19
2	29	2012.06.01	2012.08.01	2012.11.30	2012.06.01	2012.11.30	2013.12.21
3	25	2012.06.01	2012.08.18	2012.12.15	2012.06.01	2012.12.15	2013.12.19
4	19	2012.06.01	2012.08.04	2012.12.08	2012.06.01	2012.12.08	2013.12.16
5	35	2012.06.04	2012.08.02	2012.11.30	2012.06.04	2012.11.30	2013.12.19
6	24	2012.06.06	2012.08.10	2012.12.15	2012.06.06	2012.12.15	2013.12.19
7	19	2012.06.21	2012.08.27	2012.12.29	2012.06.21	2012.12.29	2013.12.19
8	22	2012.06.23	2012.08.31	2012.12.29	2012.06.23	2012.12.29	2013.12.21
9	22	2012.06.30	2012.08.28	2012.12.25	2012.06.30	2012.12.25	2013.12.19
10	18	2012.07.17	2012.09.18	2013.02.26	2012.07.17	2013.02.26	2014.02.05
11	21	2012.07.26	2012.09.28	2013.02.01	2012.07.26	2013.02.01	2014.02.06
12	25	2012.08.25	2012.10.27	2013.03.06	2012.08.25	2013.03.06	2014.03.27
13	18	2012.08.28	2012.10.26	2013.02.28	2012.08.28	2013.02.28	2014.03.24
14	16	2012.08.24	2012.10.30	2013.03.07	2012.08.24	2013.03.07	2014.03.17
15	24	2012.09.04	2012.11.07	2013.02.25	2012.09.04	2013.02.25	2014.02.28
16	25	2012.09.20	2012.11.24	2013.03.25	2012.09.20	2013.03.25	2014.03.25
17	24	2012.10.05	2012.12.01	2013.04.27	2012.10.05	2013.04.27	2014.04.16
18	20	2012.12.06	2013.02.19	NA	2012.12.06	NA	NA
19	31	2012.12.15	2013.02.09	2013.06.08	2012.12.15	2013.06.08	2014.07.22
20	19	2012.12.17	2013.02.20	2013.06.24	2012.12.17	2013.06.24	2014.07.29

* Aged 12 to 35 (22.4 ± 5.3 years old).

NA, not applicable due to the withdrawal.

All women were vaccinated with Gardasil^®^.

We measured the concentration of these sera by MB-ELISA and compensated for varying values among plates by using an in-house reference sample. We then plotted converted OD values with the WHO international unit (IU/mL) as described in Materials and Methods and in **S2B Fig**. To assess the correlation between HPV-16 and HPV-18, both values from the same individual were plotted in a scatterplot analysis graph (**Fig 2D** and **2E**). We observed significant correlation between HPV-16 and HPV-18 at the first time point (Pearson’s *r* = 0.8492; p < 0.0001) and at the second time point (Pearson’s *r* = 0.9092; p < 0.0001), suggesting parallel effects of each antigen on the clinically used vaccines.

### Measurement of serum anti-HPV-16 L1 and anti-HPV-18 L1 Ab levels in clinical settings

We next validated the increased serum levels of anti-HPV Abs in vaccinated individuals from other hospitals in addition to the samples, which included patients with cervical cancer and cervical cancer-related diseases. We examined the collected samples consisting of 6 groups including cervical cancer and cervical cancer-related entities in addition to another group of vaccinated individuals (**Table 2**).

**Table 2:** List of cervical cancer-related disease groups and control groups.

Group No.	Category	Age		Number
		Range	Mean ± SD	
1	Children	3–16	9.56 ± 4.2	18
2	Not vaccinated	19–38	30.6 ± 5.9	25
3	Vaccinated	12–44	22.1 ± 6.7	113
4	CIN 1/CIN 2	28–63	40.8 ± 12	9
5	CIN 3	21–61	39.1 ± 12	7
6	Cervical cancer	39–77	55.6 ± 14	17

CIN 1–3; cervical intraepithelial neoplasia grade 1–3.

**Fig 3:**
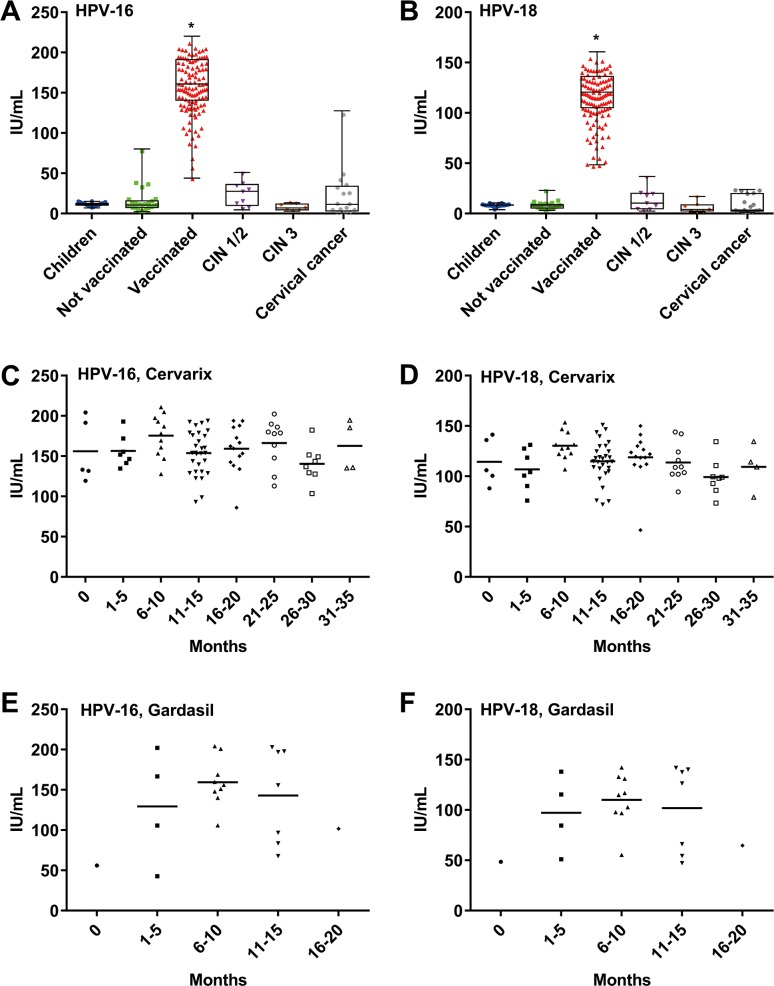
Comparison of the serum levels of HPV-16/-18 Abs in healthy and clinically diagnosed groups. The levels of HPV-16/-18 Abs were measured using serum samples from children (Children), unvaccinated healthy women (Not-vaccinated), and vaccinated healthy women (Vaccinated), as well as from patients diagnosed as having cervical intraepithelial neoplasia grade 1 or 2 (CIN 1/2), CIN 3, or cervical cancer. **A and B.** Comparison of anti-HPV-16/-18 Ab concentrations in the sera of each category. Asterisk (*) represents p < 0.001, one-way ANOVA. **C–F.** Change in serum HPV-16/-18 Ab concentration at the indicated times post vaccination with Cervarix (**C and D**) and Gardasil (**E and F**).

The concentrations of serum anti-HPV-16 and anti-HPV-18 Abs were measured by MB-ELISA (**Fig 3**).

We confirmed the increase in anti-HPVs L1 Abs in the vaccinated group. Additionally, the levels of serum Ab against HPV-16 L1 was similar to that of HPV-18 L1. The levels of anti-HPVs L1 Abs in children, unvaccinated, and CIN 3 individuals were low, whereas the Ab concentrations in CIN 1/CIN 2 and cervical cancer patients were slightly high. Notably, the level of Abs in the vaccinated group was highest among the 6 groups evaluated (HPV-16; 155.0 ± 34.2 IU/mL, HPV-18; 112.0 ± 24.9 IU/mL) (**Fig 3A** and **3B**). We also analyzed the elapsed time effect on the serum levels of anti-HPV-16 and anti-HPV-18 Abs in vaccinated individuals (**Fig 3C–3F**). These concentrations were kept high for more than 15 months after vaccination in both Cervarix^®^ (**Fig 3C** and **3D**, n = 91) and Gardasil^®^ (**Fig 3E** and **3F**, n = 22) vaccinated individuals. Moreover, these specific Abs were still detected after 30 months in individuals vaccinated with Cervarix^®^ (**Fig 3C** and **3D**). No statistically significant difference was observed between the experimental time groups (p > 0.01). (**Fig 3C** and **3D**).

### Correlation between the newly developed IC method and MB-ELISA

**Fig 4:**
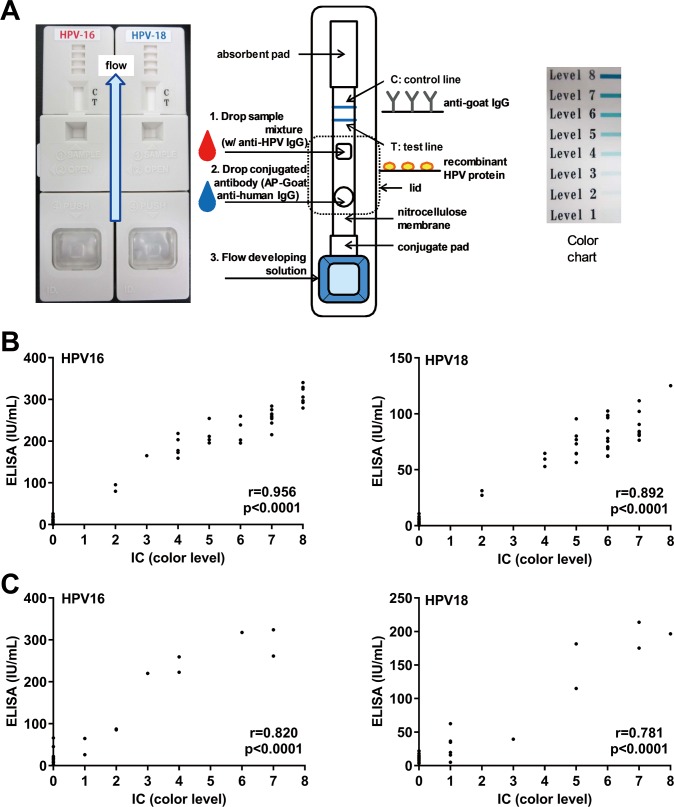
Correlation between IC and MB-ELISA. **A.** Technical details of IC method to visualize L1 Abs. IC values are given as a visual score, determined by the color chart. (C, control line; T, test line). **B and C**. Scatter plots of IC (x-axis) and MB-ELISA (y-axis) from the same samples for anti-HPV-16 and HPV-18 Ab levels. **B.** The same samples in **Fig 2** were measured by IC. **C.** The same samples in **Fig 3** were measured by IC. Five samples were arbitrarily selected from each group. Appearance of IC results were shown in **S3 Fig**.

We developed an IC method as described in the Materials and Methods section and in **Fig 4A**. To verify the consistency between IC and MB-ELISA, the IC method was applied to the same corresponding serum samples that were measured in **Figs 2** and **3**. These values were plotted in the scattered plots to compare these methods. We found significant correlation between IC and MB-ELISA in both HPV-16 L1 and HPV-18 L1 as shown in **Fig 4B** and **4C**.

These data suggest that the measurement results by our IC correctly reflect those by MB-ELISA.

## Discussion

We have developed a simple and quick IC method that enables measurement of serum anti-HPV-16/-18 L1 Abs. This IC method is single, rapid, and semi-quantitative and provides results that highly correlate with the MB-ELISA method.

Screening of cervical cancer in developed countries has been carried out since the 1960s [38], and the onset and mortality decreased significantly in these countries after starting HPV vaccination [39]. The HPV vaccine is still not delivered to a lot of developing countries, although HPV vaccination programs have started in a number of regions [40]. For retrospective evaluation, it would be worthwhile to provide cost-effective and non-laboratory environmental tools for the detection of anti-L1 Abs, especially in developed countries. Since the IC method can be implemented without any experienced techniques and electronic devices, it would be valuable as a point-of-need tool for simple and quick monitoring of the effect of the HPV vaccine [41]. Moreover, for prospective evaluation, it is suitable to monitor children with underlying medical problems including HIV carriers [42] and juvenile autoimmune diseases [43,44], especially in developing countries. Future research is needed to reveal the sufficient specific Ab titer to prevent HPV infection. Its surveillance would allow us to know the timing of boost immunization or decrease the times of vaccination.

Despite the fact that most cases in disease entities used in this study should have ongoing HPV infection in the lesion, we did not observe a significant increase in anti-HPVs L1 Ab titers in patients whom have lesions of cervical cancer or dysplasia. It has been reported that Ab titers in natural HPV infection cases are over 100 times lower than in HPV vaccinated cases [11]. Only a few samples showed a relatively high concentration of anti-HPVs L1 natural Ab in the non-vaccine group of this study, indicating that there is variability that is likely attributable to individual differences in immune responses to vaccination or HPV infection. To date, no minimum protective Ab level has been determined in the commercialized HPV vaccines with the absence of lesions with dysplastic change in vaccinated individuals. Additionally, a murine study has shown that the effective level of neutralizing Abs against HPV *in vivo* is significantly lower than those in *in vitro* neutralizing assays [45]. This implies that a relatively low titer for HPV L1 Abs might also be efficient in humans.

Interestingly, anti-HPV L1 Abs are not only detected in serum but also in other body fluids, such as oral fluid [46,47] and vaginal lavage [48,49]. These Abs are important for immune surveillance. Accordingly, it has been demonstrated that the efficacy of the HPV vaccine is due to the cervical secretion of IgG [48]. Since the increase in anti-HPV L1 neutralizing Abs in serum correlates with that in cervical secretion [50], these samples can be measured by a non-invasive alternative assay for accurate evaluation of the vaccine efficacy.

In this study, we have measured sera only from Japanese female with IC method. We should extend the IC method for male and for samples reflect ethnic diversity. Additionally, we refer that the measurement by IC method might not be suitable for the detection of HPV type-specific Abs as inferred from the results of direct ELISA that have relatively high background (Fig 1C). In that case, an IC test combining two types of HPV might be an option to concur this problem. Moreover, in terms of the future implementation of our diagnostic kit in large scale, one problem is the low-temperature transport and preservation of the kit especially in developing countries, and another is the production of recombinant HPV L1 antigens from silkworms with low cost. Our future diagnostic kit should circumvent these problems with providing solutions including long term stability under several temperature conditions.

Taken together, we developed a simple and quick IC chip for anti-HPV-16 L1 and anti-HPV-18 L1 Ab concentration measurement. With less than one drop of serum, our IC chip can easily detect serum HPV-16/-18 Ab concentration within 15 minutes, without the need for electronic devices or techniques.

## Supporting information

S1 Fig Summary of the production of recombinant L1 proteins. Synthesized L1 genes of HPV-16 and HPV-18 were subcloned into the pDock-His_pM01 vector (**A, B**). Baculoviruses obtained using the BmN cell line with BmNPV were injected into silkworm pupae. Recombinant L1 proteins were purified using the dock-tag purification system.
(TIF)

S2 Fig Immunoreactivities of control serum and WHO HPV standard L1 Abs against Cervarix antigen and conversion to the international unit. **A**). Direct ELISA showed the expected dual immunoreactivities of WHO international standard serum but not of control serum against Cervarix. Asterisk (*) represents p < 0.05, one-way ANOVA. **B**). Standard curve and calculated equation using WHO standard L1 Abs.
(TIF)

S3 Fig Appearance of IC results to detect serum HPV-16/-18 L1 Abs. Appearance of the IC for detecting HPV-16 L1 and HPV-18 L1 Abs in **Fig 4B** and **4C**. We determined the scores by direct visual observation but not by these photos.
(TIF)
