# Supplementary figures and images for: Development of a simple and quick immunochromatography method for detection of anti-HPV-16/-18 antibodies
Source: PLoS One. 2017 Feb 3;12(2):e0171314. doi: 10.1371/journal.pone.0171314 (PMC5291722; doi:10.1371/journal.pone.0171314)

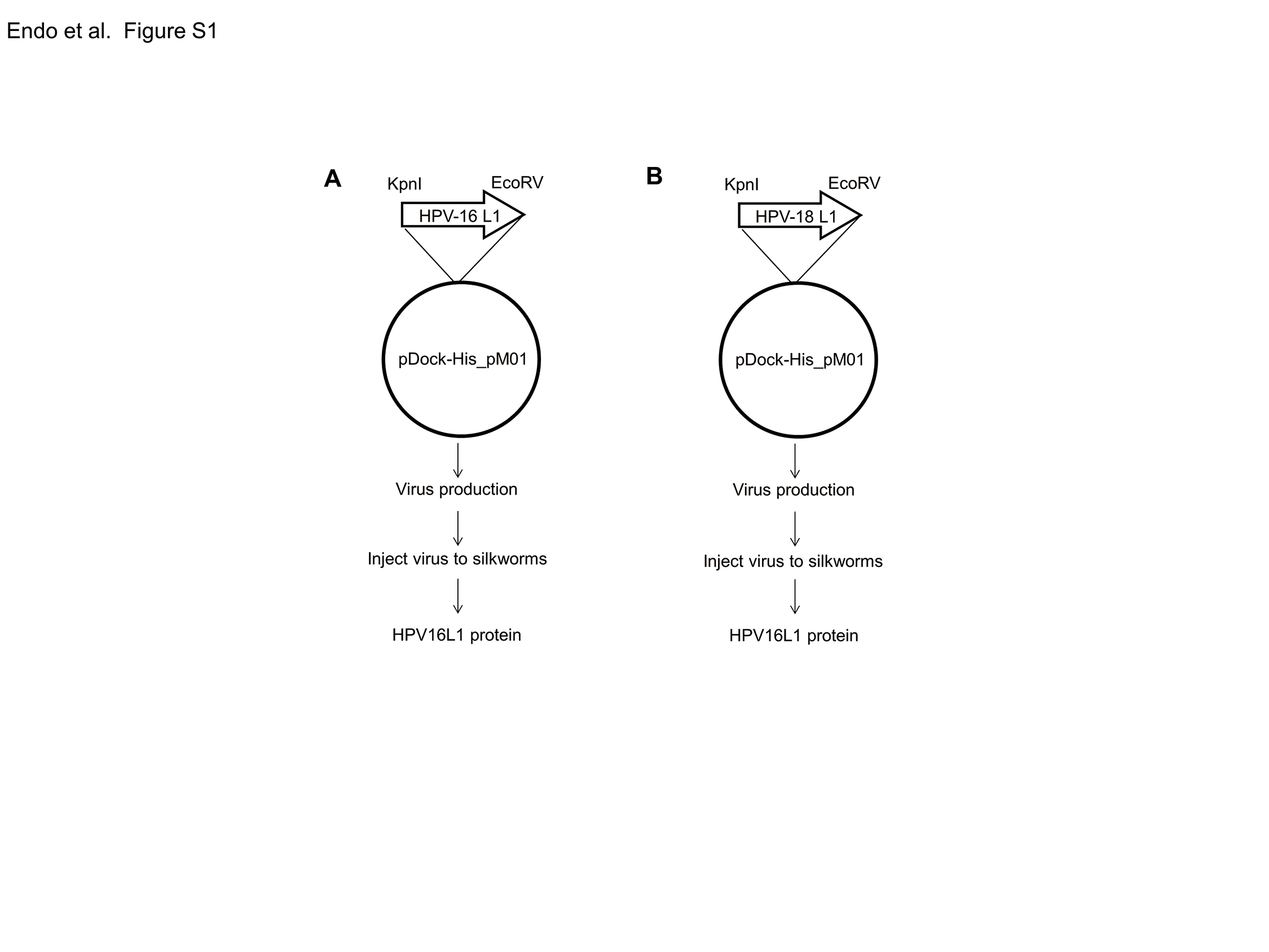

Supplement: S1 Fig — Synthesized L1 genes of HPV-16 and HPV-18 were subcloned into the pDock-His_pM01 vector (A, B). Baculoviruses obtained using the BmN cell line with BmNPV were injected into silkworm pupae. Recombinant L1 proteins were purified using the dock-tag purification system. (TIF) [file pone.0171314.s001.tif]

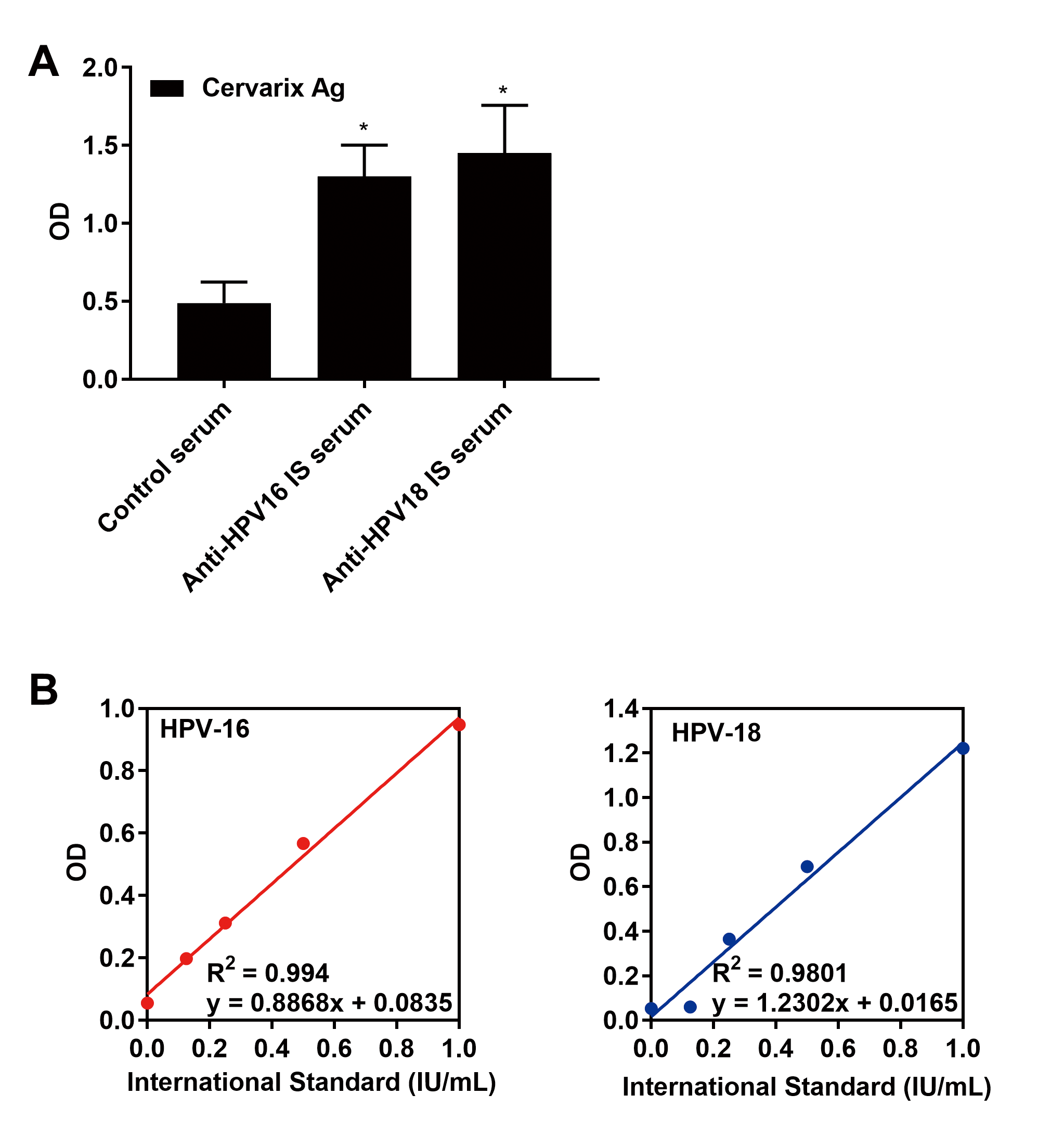

Supplement: S2 Fig — A). Direct ELISA showed the expected dual immunoreactivities of WHO international standard serum but not of control serum against Cervarix. Asterisk (*) represents p < 0.05, one-way ANOVA. B). Standard curve and calculated equation using WHO standard L1 Abs. (TIF) [file pone.0171314.s002.tif]

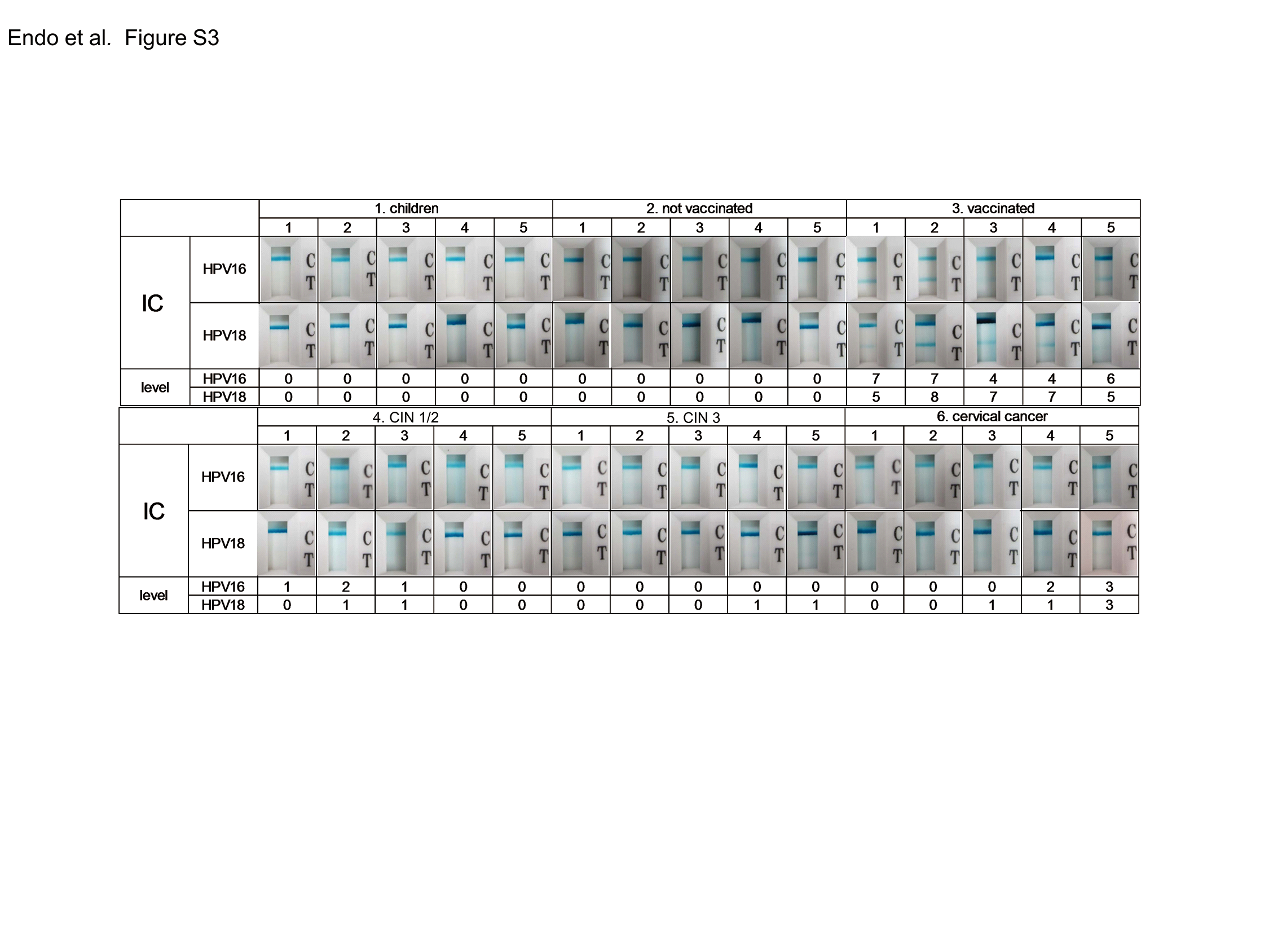

Supplement: S3 Fig — Appearance of the IC for detecting HPV-16 L1 and HPV-18 L1 Abs in Fig 4B and 4C. We determined the scores by direct visual observation but not by these photos. (TIF) [file pone.0171314.s003.tif]
